# The Multidimensional Nature of Semantic Transparency in a Cross‐Linguistic Perspective: Evidence From Human Intuitions, Computational Estimates, and Processing Data for Chinese Compounds

**DOI:** 10.1111/cogs.70194

**Published:** 2026-03-10

**Authors:** Jing Chen, Emmanuele Chersoni, Marco Marelli, Chu‐Ren Huang

**Affiliations:** ^1^ Department of Psychology University of Milano ‐ Bicocca; ^2^ Department of Language Science and Technology The Hong Kong Polytechnic University

**Keywords:** Semantic transparency, Multidimensionality, Computational estimates, Large language models, Compound processing

## Abstract

Semantic transparency is a key construct for understanding how complex words are represented and processed, yet it has been conceptualized and operationalized in diverse ways across studies. In this study, we validate whether semantic transparency exhibits multidimensional properties across different measures in Mandarin Chinese. We first construct a novel dataset consisting of 2675 nominal compounds, with a rich set of measures from human ratings, traditional distributional semantic models, and recent large language models. To investigate whether they inform the same aspects of this construct, we then examine the latent structure among these measures through exploratory factor analysis. Our factor analysis reveals that this construct is fundamentally multidimensional, with measures assessing the semantic contribution of each constituent and the semantic predictability of overall compounds representing distinct factors in the latent structure. These derived composite factors also predict lexical decision performance, with the factor representing second constituent contribution showing significant facilitatory effects. Our work extends the cross‐linguistic validity of the multidimensionality hypothesis of this theoretical construct previously established in English and German to Chinese compounds. Additionally, we provide a valuable resource for future research on the representation and processing of compounds, together with methodological insights into using computational estimates to augment psycholinguistic datasets across dimensions of semantic transparency.

## 1 Introduction

A fundamental question in linguistics and psychology concerns how complex words are represented in the mental lexicon (e.g., Bybee, 1995; Hampton, 1997; Schäfer, 2017; Taft & Forster, 1976; Winter & Hampton, 2017). The central debate revolves around whether these words undergo morphological decomposition to benefit from the activation of their individual constituents during lexical access (e.g., Baayen et al., 2011; Ji, Gagné, & Spalding, 2011; Schreuder & Baayen, 1995; Zwitserlood, 1994).

Within this debate, semantic transparency has emerged as a critical construct in understanding such morphological processing. It refers to the degree to which the overall meaning of a whole word can be derived from its constituents, spanning from semantically transparent (e.g., 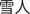, “snow + man, snowman”) to opaque (e.g., 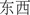, “east + west, things”). Schreuder and Baayen (1995) proposed a meta‐model of complex word processing in which semantically transparent complex words are characterized by substantial semantic overlap between the representations of the whole word and those of its constituents. Building on this framework, Libben (1998) hypothesized that transparency serves as a crucial link between representations at the lexical level (i.e., morphemes and whole words) and those at the conceptual level (i.e., concepts and their semantic features). This helps explain the processing differences between compounds like “strawberry” and “blueberry,” where “blue” maintains a conceptual link to “blueberry” but “straw” lacks such a transparent connection.

The role of semantic transparency in compound processing has been extensively studied in psycholinguistics, yet the results remain contentious (e.g., Gagné, Spalding, & Schmidtke, 2019; Günther, Marelli, & Bölte, 2020; Libben, Gibson, Yoon, & Sandra, 2003; Libben, 1998; Marelli & Luzzatti, 2012; Mok, 2009; Pollatsek & Hyönä, 2005; Zwitserlood, 1994). A prevailing view is that transparent compounds are more likely to be decomposed during processing, leading to faster response times in behavioral tasks compared to opaque compounds (e.g., Ji et al., 2011; Libben, 1998; Libben et al., 2003; Sandra, 1990; Zwitserlood, 1994). However, this picture is, to some extent, complicated by how the construct of transparency was operationalized, in addition to other confounding factors (e.g., frequency, Marelli & Luzzatti, 2012; headedness, Libben et al., 2003, and experimental design differences, Auch, Gagné, & Spalding, 2020). Recent large‐scale studies on English and German compounds suggested that different measures of semantic transparency do not necessarily capture the same aspects of this construct and may yield different effects in behavioral experiments (e.g., Günther et al., 2020; Gagné et al., 2019).

To validate the nature of multidimensionality as a cross‐linguistic phenomenon, we extend the examination to Chinese, a typologically different language, with compounding as the most productive word formation mechanism (Ceccagno & Basciano, 2007; Xing, 2006) and a writing system where semantics is encoded at the orthographic level (i.e., radicals as universal subword components directly encode semantic information. See Chou & Huang, 2010; Huang, 2009). In this study, we first construct a new dataset of semantic transparency in Chinese compounds, aimed at collecting a comprehensive set of measures from human participants and computational models. Building on this resource, we then address the multidimensional nature of compounds in the Chinese language.

### 1.1 The writing system, compounds, and semantic transparency in the Chinese language

The Chinese writing system primarily employs logographic characters to represent concepts. More than 80% of Chinese characters are composed of a semantic radical and a phonetic radical, with approximately 70% of these arranged from left to right (e.g., Lee, 2022). Semantic radicals generally provide systematic semantic cues, while phonetic radicals offer hints about pronunciation (Hsiao & Shillcock, 2006). For instance, the character 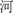 (“river,” “he2”) has two components: 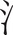 (“water”) and 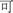 (“ke3”), indicating its semantic association with “water” and its approximate pronunciation with “ke3.” The orthographic information enables experienced readers to rapidly activate semantic information at the character level, influencing their performance in lexical processing tasks (e.g., Chen, Wang, Liu, & Liu, 2025; Hsu, Tsai, Lee, & Tzeng, 2009; Myers, 2006; Zhong, Wan, Ahrens, & Huang, 2022).

The Chinese lexicon is characterized by extensive compounding and also limited affixation (e.g., Hsieh, Hong, & Huang, 2022; Sproat & Shih, 1996). Compounding accounts for approximately 80% of common words and 90% of neologisms (e.g., Ceccagno & Basciano, 2007; Xing, 2006), making it the most productive word formation mechanism in the language. Due to limited derivational morphology, headedness is lexical‐semantically driven (Huang, Wang, & Chen, 2022; Hsieh et al., 2022), with right‐headedness predominating (Chao, 1968; Song, Xiong, Zhao, & Huang, 2022; Zhu, 1982). Following the percolation principle that the head constituent projects its information to the larger unit that it forms (Song et al., 2022; Zwicky, 1985), the second constituent determines the grammatical category and core meaning of right‐headed compounds (e.g., 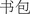, “book + bag, school bag”). Compounds with one constituent functioning as the head are termed *endocentric* compounds. In contrast, *exocentric* compounds have no head constituent, and their meanings cannot be directly predicted from the individual constituents (e.g., 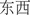, “east + west, things”).

The morphological processing of Chinese compounds has also been discussed through the lens of semantic transparency (e.g., Myers, 2006; Tse et al., 2017), but with mixed results. In a pioneering study, Mok (2009) collected transparency ratings for 192 disyllabic compounds based on dictionary definitions and human judgments on the semantic relatedness of each constituent to the compound, with each item rated by 30 participants. The author found that opaque compounds behaved like monomorphemic words (i.e., processed as whole words), while transparent compounds showed evidence of constituent activation during recognition. Building on this work, Tse et al. (2017) collected transparency ratings for 25,286 traditional Chinese disyllabic compounds from 20 participants, and found that lexical decision was faster and more accurate for compounds with higher transparency ratings for both constituents. In contrast, other studies have reported no significant differences between transparent and opaque compounds in lexical decision times (e.g., Myers, Libben, & Derwing, 2004). In addition, a linguistic analysis from Wang, Huang, Yao, and Chan (2019) suggested that endocentric compounds tend to have a more transparent head constituent (i.e., higher ratings). These mixed findings highlight the need for a more systematic investigation of semantic transparency in Chinese compounds.

### 1.2 Semantic transparency, measurements, and multidimensionality

The construct of semantic transparency has been viewed as an independent variable in psycholinguistic studies but has been conceptualized in at least two different ways in the literature: as a *constituent‐wise* or *compound‐wise* property. The former assesses how much each constituent as an individual concept relates to the compound meaning. For example, this view asks how much do “snow” and “ball” individually contribute to the meaning of “snowball” (Kim, Yap, & Goh, 2019; Mok, 2009; Tse et al., 2017). In contrast, the latter evaluates the overall predictability of the compound meaning from the combination of its constituents, such as the extent to which “snowball” can be predicted from the meanings “snow” and “ball” (Juhasz, Lai, & Woodcock, 2015; Ji et al., 2011; Libben et al., 2003; Marelli & Luzzatti, 2012; Marelli, Dinu, Zamparelli, & Baroni, 2015).

Methodologically, both views of semantic transparency can be operationalized through human ratings or computational estimates. Human ratings are typically collected by explicitly querying (e.g., Gagné et al., 2019; Juhasz et al., 2015; Kim et al., 2019; Mok, 2009; Tse et al., 2017). Computational estimates, in contrast, are primarily derived from Distributional Semantics Models (DSMs; Günther, Rinaldi, & Marelli, 2019; Lenci & Littell, 2008; Turney & Pantel, 2010), where concepts are represented as high‐dimensional vectors based on their co‐occurrence patterns in large textual corpora. For example, the commonly used *relatedness‐based measures* calculate semantic relatedness between vectors for compounds and their constituents, treating each as an isolated lexical unit corresponding to individual concepts in semantic memory (Gagné et al., 2019; Günther et al., 2019; Kim et al., 2019; Wang, Hsu, Tien, & Pomplun, 2014). By contrast, *composition‐based measures* simulate the process of combining constituents into compounds (e.g., Günther et al., 2020; Hsieh, Marelli, & Rastle, 2025; Marelli, Gagné, & Spalding, 2017; Wang & Xu, 2025), taking the morphological roles of constituents into account using compositional DSMs (e.g., Baroni, Bernardi, & Zamparelli, 2014; Lenci, Sahlgren, Jeuniaux, Cuba Gyllensten, & Miliani, 2023).

This naturally raises the question of whether these two views, together with their varied operationalizations, have equivalent predictive power for transparency effects in lexical processing tasks. Juhasz et al. (2015) collected compound‐level transparency ratings for 629 English compounds and found facilitatory effects in lexical decision times but not word naming latencies based on data from the English Lexicon Project (i.e., ELP, Balota et al., 2007). Later, Kim et al. (2019) found that their newly collected constituent‐level ratings (i.e., semantic relatedness between each constituent and the compound meaning) predicted both lexical decision and naming data from ELP, accounting for more variance than the compound‐level ratings from Juhasz et al. (2015) and the relatedness‐based estimates from Mandera, Keuleers, and Brysbaert (2017). More recently, Gagné et al. (2019) observed even more mixed patterns: their compound‐level ratings and first constituent contribution predicted lexical decision data from both ELP and the British Lexicon Project (i.e., BLP, Keuleers, Lacey, Rastle, & Brysbaert, 2012), while the second constituent contribution only successfully predicted BLP performance. Additionally, they found that *relatedness‐based measures* derived from SNAUT vectors (Mandera et al., 2017) were less predictive than those extracted from Latent Semantic Analysis (Landauer & Dumais, 1997).

These varied predictions of transparency effects raise the question of whether these measures necessarily instantiate the same theoretical construct. Günther et al. (2020) found that *composition‐based* estimates of German compounds effectively captured facilitatory effects in lexical decision data, with higher modifier composition predicting faster response times (also see Günther & Marelli, 2019). In contrast, *relatedness‐based estimates* better captured transparency effects in an eye‐tracking study where participants judged whether two words were semantically related. They proposed that relatedness‐based measures may better predict performance in tasks requiring access to whole‐word compound semantics, while composition‐based measures may be more suited to tasks demanding faster access, reflecting the ease of combining constituents. They thus argued that semantic transparency is a multidimensional construct, and that single measures may provide an incomplete picture of this complex phenomenon.

Auch et al. (2020) further advanced the multidimensional hypothesis by explicitly examining the latent dimensions behind this variety of operationalizations. They considered 11 common transparency measures using data from Kim et al. (2019) and Gagné et al. (2019), which included both constituent‐ and compound‐level human ratings, as well as relatedness‐based computational estimates measuring semantic similarity between each constituent and the compound, and between the two constituents. Their exploratory factor analysis revealed that these measures captured at least four distinct aspects of the construct: (1) human ratings of first constituent contribution; (2) computational estimates and human ratings of second constituent contribution; (3) computational association between compounds and both constituents; and (4) semantic association between the two constituents. After excluding the fourth factor due to its limited predictive power, they reanalyzed the data using a two‐factor model, which revealed that constituent position (first vs. second), rather than measurement type (human rating vs. computational estimate), fundamentally distinguished the factors. Critically, these estimated factors, which captured the latent structure of semantic transparency, differentially predicted lexical decision and naming data from both ELP and BLP, with the factor representing the second constituent contribution showing the most robust effects across all response time measures. This analysis thus provided strong evidence for the multidimensionality hypothesis of semantic transparency in English compounds.

### 1.3 The present study

In the present study, we extend the investigation of the multidimensionality hypothesis to a typologically different language, Mandarin Chinese.

First, we provide a transparency dataset with a rich set of measures from both constituent‐wise and compound‐wise theoretical views of this construct (e.g., Gagné et al., 2019). We reflected these views through both human ratings and computational models, and computational estimates include both relatedness‐based and composition‐based measures. Since recent work demonstrates that Large Language Models (henceforth LLMs) judges show remarkable alignment with human performance in linguistic and psycholinguistic tasks (Brysbaert, Martïnez, & Reviriego, 2024; Brysbaert, Martínez, & Reviriego, 2025; Martínez, Conde, Reviriego, & Brysbaert, 2024; Martínez et al., 2024; Peng, Hsu, Chersoni, Qiu, & Huang, 2025; Trott, 2024) and outperform traditional frequency‐based estimates in predicting behavioral data (Brysbaert et al., 2025), we include LLM‐generated transparency ratings as a novel computational measure. Given that word length may influence transparency (e.g., Günther et al., 2020; Gagné et al., 2019; Kuperman, Schreuder, Bertram, & Baayen, 2009), we extend transparency investigation beyond disyllabic words, which is the primary focus of previous Chinese research (Mok, 2009; Tse et al., 2017; Wang et al., 2019), to include trisyllabic and tetrasyllabic compounds.

Building on this dataset, we examine whether these measures capture a unified latent structure, validate whether the multidimensional nature of semantic transparency extends to Chinese, and assess their predictive power for lexical decision data (MELD‐SCH; Tsang et al., 2018).

## 2 Study 1: Human ratings on semantic transparency

### 2.1 Participants

Twenty native Mandarin speakers (15 female, 5 male; mean age = 28 years) participated in the rating task. They were recruited through Chinese social media platforms (WeChat and QQ) and were affiliated with several academic institutions, including Peking University, Leiden University, The Hong Kong Polytechnic University, and Shanghai Ocean University. Ten participants had a major in Chinese Linguistics, while the remaining 10 came from other disciplines. Ethical approval was obtained from the Research Ethics Committee at The Hong Kong Polytechnic University (Reference Number: HSEARS20220407007‐02). Participants provided their informed consent and were compensated for their participation.

### 2.2 Word materials

Our dataset includes 2675 Chinese nominal compounds drawn from two resources: Zhou, Chersoni, and Hsu (2026) and Wang et al. (2019).^1^ Specifically, these compounds are noun‐noun compounds (NNCs), a particularly interesting subtype consisting of two nominal constituents, resulting in a homogeneous morphological structure but diverse implicit semantic relations between constituents (Gagné & Spalding, 2004; Nakov, 2013; Rambelli, Chersoni, Collacciani, & Bolognesi, 2024; Schäfer, 2017; Wang, Huang, Yu, & Li, 2010).

Among them, 2088 NNCs were sourced from Zhou et al. (2026), who annotated semantic relations between nominal constituents in compounds extracted from the newspaper *People's Daily*.^2^ These compounds were identified through automated processing using the *Jieba* package for word segmentation and part‐of‐speech tagging to extract nominal units from the corpus. Five native Mandarin speakers then validated the resulting candidate NNCs and reached consensus on the final selection. We supplemented this wordlist with all NNCs from Wang et al. (2019). After removing duplicates, the combined list contained 2675 unique NNCs covering three different lengths: 1003 disyllabic NNCs (e.g., 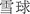 “snowball”), 275 trisyllabic NNCs (e.g.,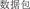 “data packet”), and 1397 tetrasyllabic NNCs (e.g., 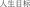 “life goal”). Each NNC was rated by all 20 participants.

### 2.3 Procedures

We collected semantic transparency ratings through both *constituent contribution* and *overall predictability*. Participants rated each target word using a scale from 0 (not at all) to 5 (greatly) to the following questions, yielding three distinct rating variables per compound:

1.

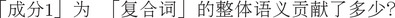
 *To what extent does [constituent 1] contribute to the meaning of the [compound]*? (ratingC1) ^3^
2.

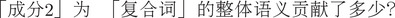
 *To what extent does [constituent 2] contribute to the meaning of the [compound]?* (ratingC2)
3.

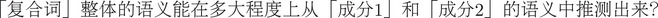
 *To what extent is the meaning of the [compound] predictable from the meanings of [constituent 1] and [constituent 2]?* (ratingCOMP)

Given the potential association between semantic transparency and headedness (e.g., Libben et al., 2003; Marelli & Luzzatti, 2012; Wang et al., 2019), participants were also asked to indicate their intuition about the head of each NNC by selecting one of the three options: (1) the first constituent; (2) the second constituent; or (3) neither.^4^ Participants were instructed to select the third option if neither constituent functioned as the head.

The surveys were administered through *Microsoft Forms*, with each survey containing 25 randomly sampled NNCs. After completing all four questions (three semantic transparency questions plus headedness) for one NNC, participants could advance to the a new page. The surveys were self‐paced, allowing participants to pause and resume at their convenience.

### 2.4 Results

#### 2.4.1 Validity and reliability

We first validated our rating data against Wang et al. (2019), who collected transparency ratings on both constituent‐level and compound‐level transparency, using the set of common targets (*N* = 664). The results show strong positive Spearman correlations across all three variables (all *p* < .001), despite slight differences in rating scales and survey design: compound‐level transparency (ρ = 0.816), first constituent contribution (ρ = 0.771), and second constituent contribution (ρ = 0.799).^5^

We also assessed inter‐rater agreement among the 20 participants using *Krippendorff's alpha* (Krippendorff, 2018), which yielded scores of 0.844, 0.837, and 0.844 for ratingC1, ratingC2, and ratingCOMP, respectively. Notably, we observed a slightly higher agreement among participants with a background of Chinese linguistics (0.833, 0.837, 0.880), relative to the rest from other disciplines (0.800, 0.799, 0.797). Nevertheless, the inter‐rater reliability remains comparable to the scores reported in previous studies, which range from 0.76 to 0.93 (Gagné et al., 2019; Kim et al., 2019; Tse et al., 2017).

#### 2.4.2 Descriptive statistics of semantic transparency ratings

**Fig. 1:**
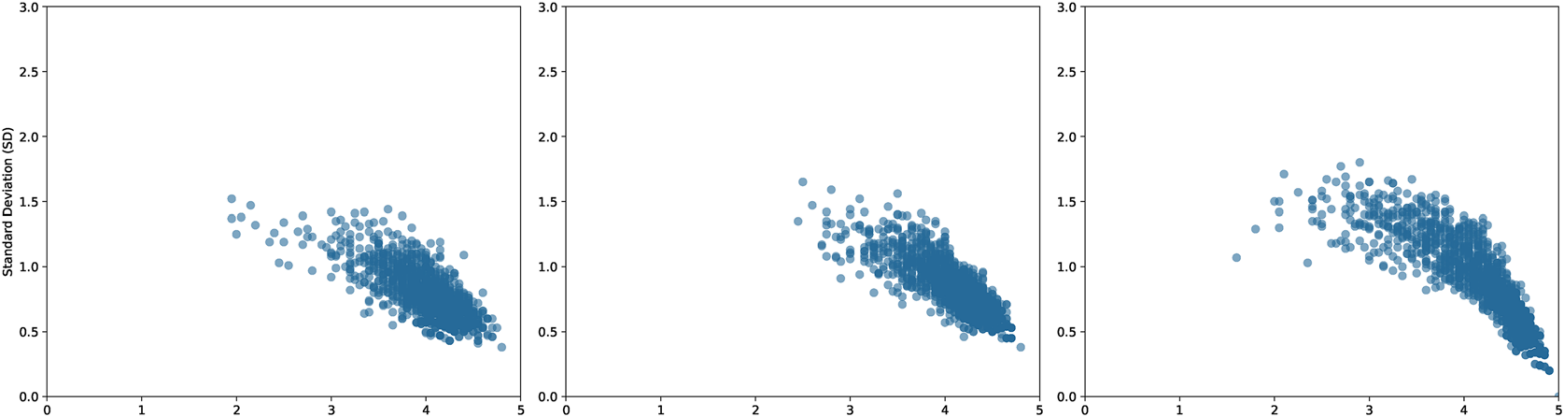
Distribution of mean ratings (x‐axis) and standard deviations (y‐axis) for ratingC1 (left), ratingC2 (middle), and ratingCOMP (right).

The semantic transparency ratings were averaged across the 20 participants for each NNC, resulting in three mean ratings per compound: ratingC1, ratingC2, and ratingCOMP. As shown in Table 1 and Fig. 1, the distribution is skewed toward moderate to high transparency, with most ratings clustering between 3 and 5. Mean ratings exceeded 4 across the three dimensions, indicating that the majority of compounds are perceived as relatively transparent. Moreover, higher ratings (4–5) typically correspond to lower standard deviations (SDs), indicating greater consistency among participants. In contrast, compounds rated as moderately transparent (2–3) show higher SDs, indicating greater variability in participant judgments.

**Table 1:** Descriptive statistics for human ratings on semantic transparency

		*N*	Mean	SD	Min	Max
all items						
	ratingC1	2675	4.11	0.71	1.95	4.80
	ratingC2	2675	4.20	0.76	2.45	4.80
	ratingCOMP	2675	4.33	0.74	1.60	5.00
len(comp) = 2						
	ratingC1	1003	3.97	0.80	1.95	4.80
	ratingC2	1003	3.99	0.88	2.45	4.65
	ratingCOMP	1003	4.04	0.93	1.60	4.85
len(comp) = 3						
	ratingC1	275	4.19	0.67	3.05	4.70
	ratingC2	275	4.27	0.73	3.55	4.70
	ratingCOMP	275	4.46	0.68	3.25	4.90
len(comp) = 4						
	ratingC1	1397	4.20	0.65	3.20	4.70
	ratingC2	1397	4.33	0.68	3.54	4.80
	ratingCOMP	1397	4.51	0.62	2.25	5.00

*Note*: *N* refers to the number of responses collected. Mean, SD, Min, Max = mean, standard deviation, minimum, and maximum of ratings. Len(comp) = the length of the compound.

Compound‐level transparency (ratingCOMP) showed the highest overall scores (*M* = 4.33, *SD* = 0.74, range: 1.60–5.00). The second constituent consistently received higher transparency ratings than the first constituent across all compound lengths. All three measures demonstrated similar patterns by length: transparency increased with compound length, while variability was greatest for disyllabic compounds.

These length‐based differences were statistically significant based on Kruskal–Wallis tests, with the most pronounced effects observed for compound‐level transparency (ratingC1: H = 324.864, ratingC2: H = 591.035, ratingCOMP: H = 715.763; all p<.001). Post‐hoc analyses using Dunn's tests (Dinno, 2015; McKight & Najab, 2010) indicated that disyllabic NNCs differed significantly from longer NNCs across all three rating dimensions (p<.001), while three‐ and four‐character (i.e., trisyllabic and tetrasyllabic) NNCs showed significant differences for ratingC2 (p<.001) and ratingCOMP (p<.01), but not for ratingC1 (p=.934). We discuss possible explanations for these length‐based differences in Section 2.5.

#### 2.4.3 Headedness information

The headedness information for each compound was determined based on the majority choice among the 20 participants. Overall, the database contains 2548 right‐headed compounds (e.g., 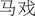 “horse + show, circus”; 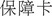 “support + card, guarantee card”), 21 left‐headed compounds (e.g., 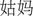 “aunt + mother, father's sister”; 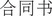 “contract + book, contract”), and 106 exocentric compounds.

These exocentric compounds were disyllabic and demonstrated three main semantic patterns: (1) metaphorical interpretations derived from the constituents (e.g., 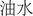, “oil + water, profit”); (2) additive meanings combining both constituents (e.g., 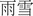, “rain + snow, rain and snow”); and (3) specialized referents not directly predictable from constituent meanings (e.g., 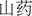, “mountain + medicine, Chinese yam”).

### 2.5 Discussion

In the analysis of human ratings across three ratings of ratingC1, ratingC2, and ratingCOMP, we observed a general trend of increasing semantic transparency with compound length, accompanied by decreasing variability in ratings.

One possible explanation for such variation at different compound lengths lies in the headedness distribution of compounds in our database. As previously reported in Section 2.4.3, disyllabic NNCs showed the greatest variability in headedness types, including right‐headed (877), left‐headed (20), and exocentric compounds (106). This variation in headedness contributes directly to differences in semantic transparency, given that the head typically projects its categorical and semantic information to the compound (Song et al., 2022; Zwicky, 1985). Exocentric compounds, in particular, tend to be more opaque and unpredictable in meaning. For example, 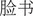 (“face + book”) refers to “Facebook,” yet its overall meaning cannot be directly inferred from its constituents. In contrast, longer NNCs exhibit near‐uniform right‐headedness, with only one left‐headed exception (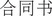, “contract + book, contract”) and no exocentric compounds. In these longer compounds, the final constituent consistently determines the overall meaning, which likely enhances semantic transparency and reduces variability in ratings.

Another factor potentially contributing to the length‐related differences is variation in the constituent structure. While the constituents of compounds function as perceptual units at the semantic level (Lee, 2022), the monomorphemic constituents, such as those of two‐character NNCs (e.g., 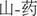, “Chinese yam”), were perceived as more ambiguous than the bimorphemic constituents primarily seen in three‐ and four‐character NNCs (e.g., 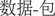, “data + bag, data packet”; 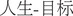, “life + goal, life goal”). This greater ambiguity primarily stems from the fact that single characters in Mandarin often carry multiple meanings due to homophony and polysemy, which can introduce greater difficulty when considering their exact semantic contribution to the compound. While participants were instructed to consider their first intuition, characters with multiple senses may still lead to increased variability and even lower transparency ratings. Tsang and Chen (2013) found that different senses of polysemous characters (e.g., 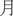, “moon,” “month,” or opaque sense in compounds such as 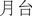 “railway platform”) are activated early during word recognition and take time to select the appropriate interpretation.

In contrast, two‐character constituents in longer compounds tend to be more semantically cohesive and less ambiguous. In addition, the structural differences between three‐ (e.g., one bimorphemic unit paired with one single character) and four‐character (e.g., two bimorphemic constituents) NNCs may further explain the rating differences in the C2 and COMP dimensions: the increased head length in four‐character NNCs potentially disambiguates or specifies semantic information contributed by the second constituent, explaining the higher transparency but lower variability in ratingC2 and ratingCOMP dimensions, compared to three‐character NNCs.

## 3 Study 2: Computational estimates of semantic transparency

This section provides computational estimates of semantic transparency for each compound using two common approaches: relatedness‐based and composition‐based measures, alongside a new paradigm of collecting LLM judges (e.g., Brysbaert et al., 2024; Martínez et al., 2024; Peng et al., 2025). To minimize potential artifacts from any single model (e.g., Kim et al., 2019), we implement these measures across multiple underlying semantic spaces. We compare these computational estimates and investigate which measures offer better simulations to human ratings that can be potentially scaled to large vocabularies and nonattested words in the future.

### 3.1 Relatedness‐based measures: Compounds as standalone concepts

Relatedness‐based measures calculate cosine similarities between vectors for constituents and compounds, treating each as a standalone concept in the lexicon rather than considering their morphological relations (e.g., 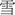 “snow” vs. 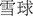 “snowball”). We exploited three pretrained models to obtain these estimates, including *fastText* (Bojanowski, Grave, Joulin, & Mikolov, 2017) and vectors from *the Tencent AI Lab Embedding Corpus for Chinese Words and Phrases* (Song, Shi, Li, & Zhang, 2018), and *BERT* (Devlin, Chang, Lee, & Toutanova, 2019). These models were selected for their extensive training, which likely ensures high‐quality representations of semantic and conceptual information.

#### 3.1.1 Models

##### Tencent AI Lab embedding corpus for Chinese words and phrases

Developed by Tencent AI Lab, this corpus provides 200‐dimensional vectors for more than 8 million Chinese words and phrases derived from sources including *Tencent News* and web pages. The vectors were trained using the Directional Skip‐Gram (DSG) algorithm, an enhancement of the traditional Skip‐Gram method that accounts for the relative positions of word pairs within text windows (Song et al., 2018). This resource offers extensive lexical coverage and semantically rich representations of words and phrases.^6^ For our analysis, we retrieved vectors for compounds and their constituents that are both present in the light version of the corpus, which contains 200‐dimensional vectors for 134,613 words and phrases.^7^ We used this version as it is publicly accessible.

##### The pretrained fastText model

The fastText model was trained on *Common Crawl* and *Wikipedia* data, and, importantly, offering a strategy to represent words including out‐of‐vocabulary (OOV) words as the sum of their subword n‐gram vectors (Bojanowski et al., 2017). For our analysis, we employed the pretrained Chinese fastText model with 300‐dimensional vectors (Grave, Bojanowski, Gupta, Joulin, & Mikolov, 2018).^8^ The model tokenized unsegmented data using the *Stanford Word Segmenter* (Chang, Galley, & Manning, 2008) to establish the in‐vocabulary word set. While the documentation does not explicitly detail the n‐gram implementation for Chinese (Bojanowski et al., 2017; Grave et al., 2018), the model likely used the default n‐gram sizes (3–6 characters for Mandarin), originally designed for alphabetic languages such as English, to infer representations for OOV words. We decided to restrict our analysis to in‐vocabulary words to ensure high‐quality representations, although the model can infer representations for disyllabic and polysyllabic OOV words in Mandarin.

It is worth noting that disyllabic words are a dominant word type in Mandarin, though their reported prevalence varies by frequency measure and genre. In terms of usage, the token frequency of disyllabic words is generally reported to be above 40%, typically remaining slightly lower than that of monosyllabic words (Huang et al., 2022). However, type frequency, that is, the percentage of unique words in a lexicon, varies significantly across corpora: approximately 46% in written‐based databases (Cai & Brysbaert, 2010; Huang & Chang, 2004), while reaching 60.98% in spoken‐language corpora (Zhao & Lei, 2025) and as high as 71.43% in curated lexica of common words (Lexicon of Common Words in Contemporary Chinese Research Team, 2008).

##### BERT

Unlike previous models that encode fixed word representations, BERT (*Bidirectional Encoder Representations from Transformers*) dynamically incorporates contextual information from both directions through multilayered self‐attention mechanisms (Devlin et al., 2019; Vaswani et al., 2017). Research suggests that syntactic information is most prominent in the earlier (encoding, for example, linear word order) and middle layers (e.g., syntactic trees, subject‐verb agreement), while more abstract semantic information appears at higher layers (e.g., semantic roles, implicit semantic relations) (Ciapparelli, Zarbo, & Marelli, 2025; Goldberg, 2019; Liu, Gardner, Belinkov, Peters, & Smith, 2019; Rogers, Kovaleva, & Rumshisky, 2020; Tenney, Das, & Pavlick, 2019). However, type‐level lexical semantic information (i.e., semantic similarity, word analogy) is distributed across multiple layers, particularly in lower ones (Vulić, Ponti, Litschko, Glavaš, & Korhonen, 2020). Recently, Miletic and Schulte im Walde (2023) suggested that lower layers in BERT may encode more information on semantic compositionality based on 280 English noun compounds, while Buijtelaar and Pezzelle (2023) found that BERT can moderately predict semantic transparency ratings from Juhasz (2018), with best performance in middle to higher layers. However, these findings were based on English compounds and estimated certain dimensions of semantic transparency, leaving it unclear whether similar patterns hold for Chinese compounds and how different layers in BERT contribute to semantic transparency estimates across dimensions.

For our analysis, we utilized bert‐base‐chinese, a 12‐layer model with 768‐dimensional representations using the Hugging Face implementation (Wolf et al., 2020).^9^ We first randomly sampled 10 sentences (each 10–30 characters long) for each compound and its constituents from a recent Chinese Wikipedia dump (zhwiki‐20241101).^10^ Each sampled sentence was tokenized using WordPiece (Wu et al., 2016), which segments Chinese text into individual characters, and then outputs an individual representation for each character. For multicharacter constituents and compounds, we applied mean pooling over characters to obtain representations in each sampled sentence. We obtained a final BERT representation for each constituent and compound by averaging context‐dependent embeddings retrieved from sampled sentences.^11^ To probe for optimal layer configurations for semantic transparency in Mandarin, we retrieved outputs from different hidden layers individually, from first to last layer.

#### 3.1.2 Measurements

We calculated cosine similarities between the target vectors obtained as described above to approximate semantic transparency, based on the assumption that semantic relatedness reflects conceptual overlap between two distinct, separate entries in semantic memory (Günther et al., 2020; Lynott & O'Donoghue, 2004; Schmidtke, Van Dyke, & Kuperman, 2018). Specifically, we implemented:

- *c1‐rel*: Cosine similarity between vectors for the first constituent and the compound.
- *c2‐rel*: Cosine similarity between vectors for the second constituent and the compound.
- *c1‐c2*: Cosine similarity between vectors for the two constituents.

### 3.2 Composition‐based measures: Compounds as compositional entities

Composition‐based measures conceptualize compounds as the result of a combinatorial process, where semantic similarities between each constituent and the resulting compound simulate the ease of integrating constituents into a combined concept (Günther et al., 2020; Gagné & Spalding, 2009). To implement this approach, we employed the theoretical framework of CAOSS (Marelli et al., 2017), which has been shown to effectively capture the compositional process for compounds across multiple studies (Günther & Marelli, 2019; Günther et al., 2020; Günther & Marelli, 2023; Hsieh et al., 2025; Wang & Xu, 2025).

#### 3.2.1 The framework: The CAOSS model

The CAOSS model views compounding as a *function application* process, where isolated words (e.g., 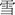 “snow”) are first transformed into role‐dependent constituents based on their morphological or positional functions (e.g., 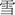 “snow” as a modifier in 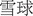 “snowball”), and subsequently combined to derive the compositional meaning of the compound (Marelli et al., 2017). This process is mathematically formalized as:

(1) c=M·u+H·v,

where *c* represents the inferred compound meaning, *u* and *v* represent vectors for two constituents as isolated words, and *M* and *H* are weight matrices encoding global information regarding different roles (i.e., positional roles or semantic roles). These matrices are learned through least‐square regression, minimizing the difference between observed compounds as isolated words in the lexicon (e.g., “carwash”) and their predicted meaning from a combinatorial process (e.g., the semantic combination of transformed “car” and “wash” according to their roles in compounding) (Günther et al., 2020; Günther & Marelli, 2023; Marelli et al., 2017).

#### 3.2.2 Semantic spaces

We retrieved vector representations for both constituents and compounds, treated as isolated words in the vocabulary, using the three distributional models introduced earlier: *Tencent AI Lab embedding corpus*, *fastText*, and *BERT*. To ensure comparability across models, we only retained NNCs and their constituents that are included in the vocabularies of all three models. This shared vocabulary consisted of 952 NNCs.

We then induced the weight matrices M and H for each model, based on the shared vocabulary. Each NNC was analyzed in terms of its constituent positional roles (i.e., *c1* and *c2*) to maintain alignment with the analyses in Sections 2 and 3.1.

#### 3.2.3 Measurements

Following Eq. 1, we computed compositional representations for each compound. We then calculated cosine similarities between the compositional representation of the compound and the relevant constituent vectors (i.e., c1‐com, c2‐com). Additionally, we computed the similarity between the compositional representation of the compound and the compound as a self‐standing word in the training corpus, following Günther et al. (2020). This latter similarity (i.e., comp‐com) reflects how well the compound meaning can be predicted from its constituents.

- *c1‐com*: Cosine similarity between the vector of the first constituent and the compositional representation of the compound.
- *c2‐com*: Cosine similarity between the vector of the second constituent and the compositional representation of the compound.
- *comp‐com*: Cosine similarity between the compound word vector and its compositional representation.

### 3.3 Prompting LLMs to predict semantic transparency

This section collected LLMs‐generated ratings from three popular LLMs: *GPT‐4* (OpenAI, 2024), *DeepSeek‐V3* (DeepSeek‐AI, 2025), and *Qwen‐plus* (Bai et al., 2023). We then evaluated their ability to simulate human‐like judgments of semantic transparency in Chinese nominal compounds.

#### 3.3.1 Procedures

We prompted each LLM with questions identical to those presented to human participants to enable direct comparison. After systematically testing several alternative formulations to optimize response consistency, we implemented the following prompt that instructed the models to provide numerical ratings on the same 0–5 scale used in our human study:

**Prompt**:

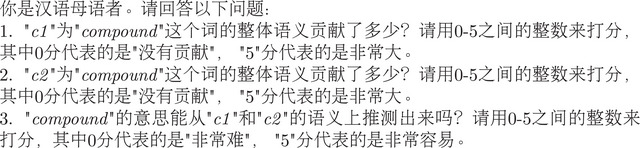

**English Translation**:

You are a native Chinese speaker. Please answer the following questions:

1. How much does *c1* contribute to the overall meaning of *compound*? Please rate using an integer between 0 and 5, where 0 represents “no contribution” and 5 indicates “very high contribution.”

2. How much does *c2* contribute to the overall meaning of *compound*? Please rate using an integer between 0 and 5, where 0 represents “no contribution” and 5 indicates “very high contribution.”

3. Can the meaning of *compound* be deduced from the meanings of *c1* and *c2*? Please rate using an integer between 0 and 5, where 0 means “very difficult” and 5 means “very easy.”

The LLMs were instructed to provide only integer scores without additional explanations or justifications, and these numerical responses were systematically formatted for subsequent analysis. In cases where models failed to provide a numerical rating or returned invalid data, we flagged such responses as −1 and excluded them from subsequent analysis.

Temperature settings significantly influence LLM response generation, with lower temperatures producing more deterministic outputs and higher temperatures yielding more diverse responses. To control response randomness, we experimented with temperatures of 0.2 and 1.0 (Gilardi, Alizadeh, & Kubli, 2023). Overall, we conducted four independent rating iterations for each LLM (two at each temperature level), where each iteration involved the complete database of 2675 NNCs.

#### 3.3.2 Measurements

For each word, LLMs provided three ratings, as identical to human ratings, in each iteration.

- *c1‐llm*: LLM‐generated ratings on the first constituent's contribution to compound meaning.
- *c2‐llm*: LLM‐generated ratings on the second constituent's contribution to compound meaning.
- *comp‐llm*: LLM‐generated ratings on the predictability of compound meaning from its constituents.

To examine the effects of temperature settings on rating consistency and accuracy, we calculated averages from two iterations at temperature 0.2, at temperature 1.0, as well as averages across four iterations, respectively, for each measure above.

### 3.4 Results and discussion

#### 3.4.1 DSMs‐derived measures

We first examine BERT layer‐wise performance in predicting human ratings of semantic transparency to determine the best‐performing layer. We then compare DSM‐generated predictions, that is, relatedness‐based and composition‐based measures, against human ratings of semantic transparency.

##### Layer‐wise analysis of BERT representations

**Fig. 2:**
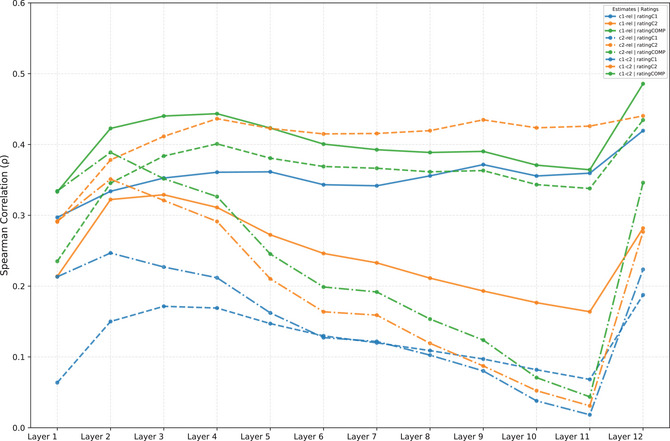
Spearman correlations between BERT layers and human ratings. Colors differentiate the three human rating dimensions. Lines differentiate computational estimates: c1‐rel, c2‐rel, and c1‐c2.

Fig. 2 presents BERT performance across the three dimensions of semantic transparency. The results show that while lower layers (except the first) generally performed well, the final layer (layer 12) achieved the highest correlations with human ratings across all dimensions: 0.486 for compound‐level transparency, 0.440 and 0.412 for constituent‐level transparency (ratingC2 and ratingC1, respectively).^12^ These findings align with previous research (Ciapparelli et al., 2025; Ormerod, del Rincón, & Devereux, 2024), suggesting that the final layer most effectively captures implicit semantic relations in compounds. However, we acknowledge that our sentence subsampling and character‐level pooling strategies may influence these results (Miletic & Schulte im Walde, 2023), warranting more systematic investigation and cross‐linguistic comparison in future work.

##### Relatedness‐ and composition‐based measures

To ensure fair comparison, the following analysis was performed on the shared vocabulary of 952 NNCs across underlying models and measures.^13^

**Fig. 3:**
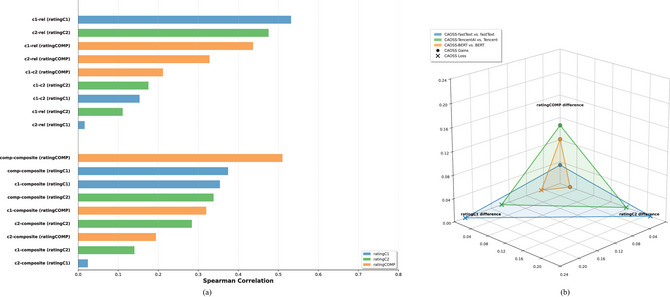
Performance comparisons across relatedness‐ and composition‐based measures. (a) Spearman correlations of measure types with human ratings, averaged across semantic models. Upper cluster: relatedness‐based measures; lower cluster: composition‐based measures. (b) Model‐specific performance improvements (CAOSS minus its counterparts) across ratingC1, ratingC2, and ratingCOMP.

Fig. 3a provides an overall comparison between relatedness‐ and composition‐based measures, averaging performance across three semantic spaces (FastText, Tencent, and BERT). The upper cluster shows that relatedness‐based measures correlate more strongly with constituent‐level transparency (with blue and green bars at the top) than with compound‐level transparency (orange bars). Conversely, the lower cluster demonstrates that composition‐based measures achieve their strongest correlations with compound‐level transparency.

Fig. 3b provides a model‐specific breakdown of performance differences. Consistent with the above patterns, all three models show improvements in predicting compound‐level transparency when using composition‐based measures, as indicated by the circles on the z‐axis (ratingCOMP). In contrast, composition‐based measures generally show decreased performance for constituent‐level transparency, marked by crosses on both the x‐axis (ratingC1) and y‐axis (ratingC2), with the exception of CAOSS‐BERT, which shows a marginal improvement of 0.02 for ratingC2.

These patterns reveal a clear functional distinction: semantic relatedness between constituents and compounds viewed as isolated lexical items effectively captures human intuitions about individual constituent contributions, but proves less effective for predicting compound‐level transparency. Conversely, composition‐based measures that explicitly model constituent interactions in specific structural positions better predict overall compound transparency.

#### 3.4.2 LLM‐generated ratings

**Fig. 4:**
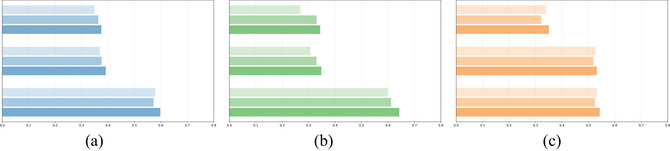
Spearman correlations between LLM‐generated ratings and human ratings. Within each subfigure, the three vertical clusters represent ratingC1 (top), ratingC2 (middle), and ratingCOMP (bottom). Color intensity indicates temperature settings: lighter, medium, darkest shades = 1.0, 0.2, and aggregated. All results are statistically significant (*p* <.001).

We reported LLM judges from both rating agreements and their correlations with human ratings. Table 2 presents intercoder agreement across LLMs at different temperature settings. In general, more deterministic outputs generated at lower temperatures (e.g., 0.2) produced more consistent responses (Krippendorff's alpha ranging from 0.686 to 0.846), in some cases approaching human‐level agreement. Also, ratings obtained at the lower temperature (0.2), compared to higher ones (1.0), generally aligned more closely with human judgments, though some variability was observed in Qwen‐plus (see Fig. 4).

**Table 2:** Krippendorff's alpha for intercoder agreements: Ratings across temperatures and models

	GPT‐4			DeepSeek‐V3			Qwen‐plus		
Temperature	c1‐llm	c2‐llm	comp‐llm	c1‐llm	c2‐llm	comp‐llm	c1‐llm	c2‐llm	comp‐llm
1.0	0.369	0.321	0.491	0.178	0.157	0.364	0.712	0.792	0.696
0.2	0.771	0.703	0.820	0.754	0.686	0.790	0.811	0.846	0.765
All	0.515	0.425	0.608	0.375	0.304	0.519	0.763	0.818	0.730

However, higher intercoder agreement among LLMs does not translate into the strongest correlations with human ratings in our study. Instead, LLM‐generated ratings aggregated across both lower and higher temperatures (indicated by the darkest shades) consistently showed stronger correlations with human ratings across all models and measures. This may be related to the composition of our human participant groups, as discussed in Section 2. Linguistic experts likely provided more deterministic and consistent evaluations (higher agreement), whereas lay participants exhibited greater variability in their responses (lower agreement). This suggests that temperature settings in LLMs could be strategically manipulated to simulate different rater populations.

#### 3.4.3 Overall comparisons

Based on the sharing vocabulary across all measures, Table 3 presents Spearman correlations between human ratings and computational predictions across all three types of measures: relatedness‐based, composition‐based, and LLM‐generated ratings. These correlations assess how well computational estimates align with human judgments of constituent‐level and compound‐level semantic transparency.^14^

**Table 3:** Spearman correlations between computational estimates and human ratings. **Bold** represents the highest correlations for each model in each dimension. Gray represents Spearman correlations were not statistically significant (*p* > .05)

Model	Measures	ratingC1	ratingC2	ratingCOMP
TencentAI	c1‐rel	**0.632**	0.085	**0.485**
	c2‐rel	−0.026	**0.642**	0.426
	c1‐c2	0.196	0.206	0.248
fastText	c1‐rel	**0.591**	0.108	**0.465**
	c2‐rel	0.007	**0.535**	0.366
	c1‐c2	0.194	0.246	0.282
BERT	c1‐rel	**0.373**	0.140	**0.360**
	c2‐rel	0.015	**0.249**	0.192
	c1‐c2	0.070	0.074	0.105
CAOSS‐Tencent	c1‐com	**0.495**	0.094	0.386
	c2‐com	−0.018	**0.488**	0.274
	comp‐com	0.421	0.414	**0.591**
CAOSS‐fastText	c1‐com	0.363	0.185	0.343
	c2‐com	0.020	0.298	0.222
	comp‐com	**0.374**	**0.328**	**0.499**
CAOSS‐BERT	c1‐com	0.204	0.143	0.230
	c2‐com	0.070	0.066	0.087
	comp‐com	**0.328**	**0.273**	**0.441**
GPT‐4	c1‐llm	0.448	−0.203	0.161
	c2‐llm	−0.061	**0.546**	0.292
	comp‐llm	**0.528**	0.370	**0.668**
DeepSeek‐V3	c1‐llm	0.454	0.021	0.324
	c2‐llm	−0.206	**0.409**	0.150
	comp‐llm	**0.546**	0.422	**0.730**
Qwen‐plus	c1‐llm	**0.455**	−0.140	0.211
	c2‐llm	−0.263	**0.541**	0.162
	comp‐llm	0.439	0.429	**0.651**

##### Compound‐level transparency

**Fig. 5:**
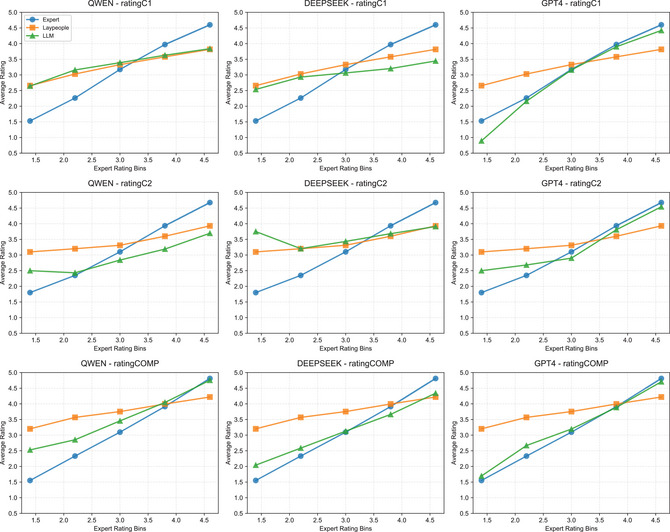
Semantic transparency estimates from experts, laypeople, and LLMs. Equal‐width bins based on expert ratings (x‐axis) show average ratings (y‐axis) for experts (blue), laypeople (orange), and LLMs (green).

LLMs substantially outperformed all other computational measures in predicting compound‐level transparency. Specifically, DeepSeek‐V3 (ρ = 0.730), GPT‐4 (ρ = 0.668), and Qwen‐plus (ρ = 0.651) achieved the highest correlations with human ratingCOMP scores. To further examine these performance patterns, we binned LLM‐generated ratings into equal‐width intervals and compared them against judgments from participants with and without Chinese Linguistics backgrounds. As shown in Fig. 5, LLMs aligned more closely with expert judgments than with lay participants, particularly for compounds in the moderate to high transparency ranges (ratings 3–5). Among the three models, GPT‐4 demonstrated the strongest overall correspondence with expert ratings across all three dimensions of semantic transparency.

Besides, composition‐based measures from the CAOSS models also showed meaningful improvements over their relatedness‐based counterparts. The *comp‐com* measure consistently outperformed corresponding relatedness measures: CAOSS‐TencentAI (ρ = 0.591) versus TencentAI (ρ = 0.485), CAOSS‐fastText (ρ = 0.499) versus fastText (ρ = 0.465), and CAOSS‐BERT (ρ = 0.441) versus BERT (ρ = 0.360). Among relatedness‐based measures, *c1‐rel* exhibited stronger correlations with compound‐level transparency ratings than either *c2‐rel* or *c1‐c2*.

##### Constituent‐level transparency

A different pattern emerged for constituent‐level transparency. DSM‐derived measures maintained clear advantages, though performance varied significantly based on the underlying semantic space. Relatedness‐based measures often outperformed other approaches: TencentAI achieved ρ = 0.632 for ratingC1 and ρ = 0.642 for ratingC2, followed by fastText (ρ = 0.591 and ρ = 0.535), while BERT showed comparatively lower correlations (ρ = 0.373 and ρ = 0.249). These strong performances came from measures comparing isolated constituents with compounds (*c1‐rel*, *c2‐rel*). In contrast, composition‐based measures generally underperformed than their relatedness‐based counterparts. CAOSS‐TencentAI yielded the best results among compositional approaches (ρ = 0.495 for ratingC1 and ρ = 0.488 for ratingC2), followed by CAOSS‐fastText (ρ = 0.374 and ρ = 0.298) and CAOSS‐BERT (ρ = 0.328 and ρ = 0.273).

LLMs demonstrated moderate correlations for constituent‐level transparency, generally surpassing composition‐based measures but falling short of the best relatedness‐based approaches. Interestingly, compound‐level LLM ratings (*comp‐llm*) often predicted first constituent contributions (ratingC1) better than the respective constituent measure (*c1‐llm*), while *c2‐llm* consistently maintained effectiveness in estimating second constituent contributions. However, for constituent‐level transparency, LLMs exhibited compressed rating distributions, assigning moderate scores to both low and high transparency items, a pattern more similar to laypeople (see Fig. 5).

In summary, these computational estimates revealed complementary strengths in aligning with human ratings: LLMs demonstrated superior performance in capturing compound‐level transparency, while traditional DSM‐based measures maintained advantages for estimating constituent‐level transparency.

## 4 Study 3: Semantic transparency as composite effectors in Chinese compound processing

The above sections have demonstrated that semantic transparency can be operationalized in multiple ways, and computational estimates showed varying degree of correlations with human ratings. In this section, we investigate whether these measures necessarily indicate the same aspects of semantic transparency by examining its latent structure (Auch et al., 2020) and their prediction in lexical decision data.

### 4.1 The latent structure of semantic transparency: An exploratory factor analysis

#### 4.1.1 Materials

Our dataset included up to 30 estimates of semantic transparency from three sources: human ratings (ratingC1, ratingC2, ratingCOMP), DSMs‐derived measures (three relatedness‐based measures and three composition‐based measures, each from three DSMs), and LLM‐generated ratings (c1‐llm, c2‐llm, comp‐llm, each from three LLMs). The subsequent analysis was based on the shared vocabulary (*N* = 952) across all measures.

#### 4.1.2 Procedures

We performed an exploratory factor analysis (EFA) to identify the latent structure of semantic transparency as reflected in these measures.^15^ Before analysis, we first conducted *Bartlett's test of sphericity* (Bartlett, 1950) and calculated the *Kaiser–Meyer–Olkin* (KMO) measure (Kaiser, 1970) to ensure data suitability and sampling adequacy.

Bartlett's test was significant (p<.001), indicating that the variables shared substantial interrelations suitable for factor analysis. Initially, with all 30 variables, the overall KMO value was 0.767. However, individual variable analysis revealed that three BERT‐related variables had inadequate sampling adequacy: *bert‐c2‐com* (Measure of Sampling Adequacy, MSA = 0.372), *bert‐c1‐com* (MSA = 0.473), and *bert‐c1‐c2* (MSA = 0.451), all below the recommended threshold of 0.5 (Lorenzo‐Seva & Ferrando, 2021). These variables were identified as noisy items that lacked discriminating power. After removing these three variables, the final data consisted of 27 variables with a substantially improved overall KMO value of 0.817, with all remaining variables achieving MSA ≥ 0.5.

**Fig. 6:**
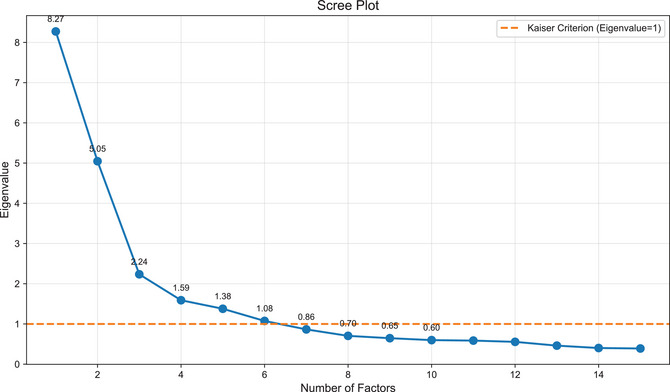
Scree plot for factors extraction: 27 measures included.

To determine the optimal number of factors to retain, we applied both the conventional eigenvalue criterion (>1.0) and scree plot analysis (Cattell & Vogelmann, 1977). Although six factors exhibited eigenvalues above 1.0, the scree plot revealed a clear elbow point between the second and third factors (see Fig. 6), suggesting a more parsimonious structure with fewer factors. To maximize interpretability, we selected a three‐factor structure and applied a *Varimax* rotation to the extracted factors.

#### 4.1.3 Results and discussion

Tables 4 and 5 present the factor loadings matrix across the three factors. We considered factor loadings exceeding 0.32 as significant, contributing to the respective factor (Auch et al., 2020; Yong & Pearce, 2013). The higher the absolute values, the stronger the contributions to that factor.

**Table 4:** Factor loadings matrix (part A)

	Human			Relatedness‐based									To be continued		
	C1	C2	CP	fastText			Tencent			BERT			fastText		
				c1‐rel	c2‐rel	c1‐c2	c1‐rel	c2‐rel	c1‐c2	c1‐rel	c2‐rel	c1‐c2	c1‐com	c2‐com	com‐p
**Factor 1**	0.72	0.52	0.82	0.38	0.08	−0.03	0.43	0.14	0.01	0.27	0.09	–	0.20	−0.08	0.46
**Factor 2**	−0.14	0.62	0.29	−0.12	0.85	0.49	−0.08	0.88	0.49	0.04	0.48	–	−0.02	0.65	0.30
**Factor 3**	0.38	−0.11	0.18	0.76	0.06	0.62	0.77	0.12	0.60	0.54	0.09	–	0.68	0.12	0.30

**Table 5:** Factor loadings matrix (part B)—continued

	Composition‐based						LLMs								
	Tencent			BERT			GPT‐4			DeepSeek			Qwen‐plus		
	c1‐com	c2‐com	com‐p	c1‐com	c2‐com	com‐p	c1	c2	comp	c1	c2	comp	c1	c2	comp
**Factor 1**	0.23	−0.05	0.55	—	—	0.34	0.41	0.28	0.83	0.52	0.13	0.84	0.44	0.07	0.79
**Factor 2**	−0.03	0.82	0.36	—	—	0.31	−0.37	0.58	0.13	−0.16	0.52	0.22	−0.31	0.69	0.22
**Factor 3**	0.80	0.15	0.35	—	—	0.38	0.43	−0.13	0.17	0.24	−0.35	0.17	0.31	−0.25	0.10

*Note*. In Tables 4 and 5, gray indicates that the measure was not significantly loaded on the factor (loading < 0.32). C1, C2, CP, com‐p, c1, c2, comp = ratingC1, ratingC2, ratingCOMP, comp‐com, c1‐llm, c2‐llm, comp‐llm. The three excluded BERT‐related measures are filled with “–.”

Specifically, Factor 1 was primarily characterized by compound‐level transparency measures, including human ratings (ratingCOMP, 0.82) and LLM‐generated ratings (comp‐llm, 0.79–0.84), along with composition‐based measures (comp‐com, 0.34–0.55). Notably, both constituent contributions showed substantial loadings on this factor: ratingC1 (0.72) and ratingC2 (0.52), alongside several computational estimates of first constituent contribution (c1‐rel, 0.38–0.43; c1‐llm, 0.41–0.52).

Factor 2 was predominantly characterized by measures assessing second constituent contribution. RatingC2 (0.62) loaded most prominently, along with strong convergence across relatedness‐ and composition‐based measures (c2‐rel, 0.48–0.88; c2‐com, 0.65–0.82). LLM‐generated ratings of second constituent contribution also loaded substantially (c2‐llm, 0.52– 0.69). In contrast, Factor 3 was dominated by DSM‐derived measures of first constituent contribution, including both relatedness‐based (c1‐rel, 0.54–0.77) and composition‐based approaches (c1‐com, 0.68–0.80). Human ratings of first constituent contribution (ratingC1) also loaded significantly on this factor (0.38), alongside interconstituent similarities (c1‐c2, 0.60– 0.62). Notably, most LLM‐generated ratings of first constituent contribution, except GPT‐4, failed to load significantly on this factor.

Given that Factors 2 and 3 distinctly relate to measures regarding the second and first constituent contributions, we interpret Factor 1 as reflecting the overall compound predictability, defined by compound‐level measures and cross‐loadings from both constituents. These cross‐loadings suggest that both constituents jointly influence overall transparency, with the first constituent showing particularly strong contributions.

**Fig. 7:**
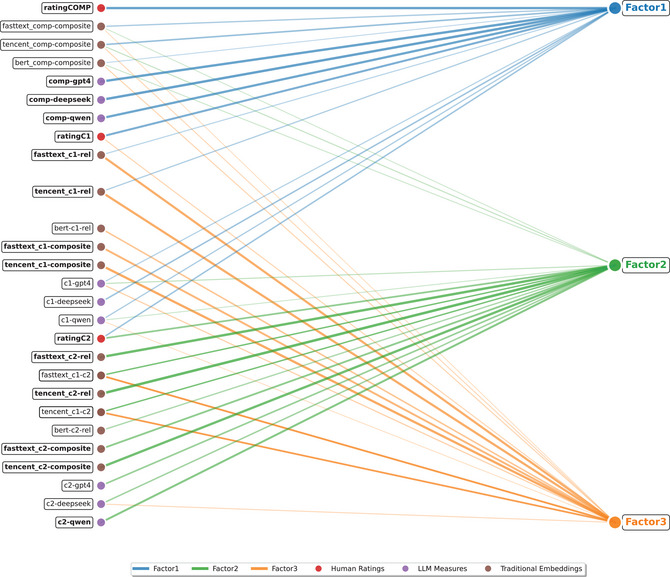
Factor analysis of semantic transparency measures. Measures in **bold** and brighter connecting lines indicate stronger factor loadings.

This three‐factor model suggests that the varied measures of semantic transparency do not necessarily capture the same aspects of the construct, with at least three distinct dimensions emerging: first constituent contribution (Factor 3), second constituent contribution (Factor 2), and overall compound predictability (Factor 1) (see Fig. 7). These findings support the multidimensional nature of semantic transparency (Auch et al., 2020; Gagné et al., 2019).

### 4.2 Predicting transparency effects from a multidimensional perspective

The latent structure of semantic transparency indicates that no single measure can fully capture the complexity of this construct. Accordingly, assessing its effects on compound processing from a multidimensional perspective may prove more informative. To this end, we computed factor scores from the three‐factor EFA model, which summarize shared variance among related measures (Auch et al., 2020) while reducing multicollinearity in subsequent regression analyses. We then examined whether these composite predictors effectively capture transparency effects in lexical decision data for Chinese compounds.

#### 4.2.1 Materials

Factor scores for each compound on the three extracted factors were computed using the regression method implemented in the *factor_analyzer* Python package. Lexical decision data from MELD‐SCH (Tsang et al., 2018) served as dependent variables, specifically standardized response times (*zRT*) and error rates (*ERR*). We included several control variables known to influence lexical processing: compound length (in characters), log‐transformed frequencies for individual constituents and whole compounds from SUBTLEX‐CH (Cai & Brysbaert, 2010), and total stroke count from Tsang et al. (2018) (e.g., Günther et al., 2020; Gagné et al., 2019; Kim et al., 2019). After excluding compounds with missing values on any variable, the final dataset comprised 308 NNCs.

#### 4.2.2 Analysis

We followed Kim et al. (2019) to perform two‐step hierarchical item‐level regression analyses to examine the predictive effects of semantic transparency factors on lexical decision data. In Step 1, we entered only lexical control variables; in Step 2, we added the three factor scores as transparency predictors. Separate models for zRT and ERR as dependent variables were run.

#### 4.2.3 Results

Table 6 presents the standardized regression coefficients from the hierarchical mixed‐effects models.

**Table 6:** Standardized zRT and error rate regression coefficients from hierarchical regression analyses for lexical decision performance

	zRT		Error rate	
Predictors	β	p	β	p
**Step 1: Lexical variables**				
Compound frequency	−.453	< .001	−.370	< .001
C1 frequency	.061	.247	.034	.540
C2 frequency	−.056	.307	−.009	.864
Compound length	−.051	.326	.022	.695
Stroke count	.007	.907	−.079	.168
$R^{2}$	.284	—	.145	—
**Step 2: + Semantic transparency**				
Compound frequency	−.447	< .001	−.365	< .001
C1 frequency	.062	.272	.020	.730
C2 frequency	−.097	.077	−.040	.472
Compound length	−.056	.278	.016	.767
Stroke count	.015	.792	−.063	.272
Factor 1	.086	.086	.019	.712
Factor 2	−.168	.001	−.136	.013
Factor 3	−.042	.447	−.061	.292
$R^{2}$	.308	—	.165	—
$\Delta R^{2}$	.024	.004	.020	.066

In Step 1, the control variables collectively accounted for 28.4% of variance in *zRT* and 14.5% in *ERR*. Among the lexical variables, compound frequency emerged as the strongest predictor, with negative standardized coefficients for both *zRT* (β=−.453, p<.001) and *ERR* (β=−.370, p<.001), indicating that higher‐frequency compounds were processed faster and more accurately. Neither constituent frequency, word length, nor stroke count significantly predicted lexical decision performance at this step.

In Step 2, the three semantic transparency factors significantly improved the *zRT* model ($\Delta R^{2}=.024$, p<.01), explaining an additional 2.4% of variance. For the *ERR* model, the factors explained an additional 2.0% of variance, but this improvement did not reach significance (p=.066). Importantly, the inclusion of these factors had minimal impact on the control variable coefficients: compound frequency maintained its strong predictive power (zRT: β=−.447, p<.001; ERR: β=−.365, p<.001), suggesting that the semantic transparency factors capture unique variance beyond lexical variables.

Among these three transparency factors, Factor 2 emerged as the only significant predictor across both dependent measures, with negative coefficients for *zRT* (β=−.168, p<.01) and *ERR* (β=−.136, p<.05), indicating that compounds with higher Factor 2 scores (i.e., where the second constituent is more transparent) were processed faster and more accurately.

## 5 General discussion

In the present study, we examined semantic transparency in Chinese compounds through various operationalizations, especially with a rich set of computational estimates. Our results revealed that commonly used measures do not necessarily capture the same aspects of this construct, supporting its multidimensional nature. We, therefore, employed factor scores that summarize shared variance among measures as composite predictors in analyzing lexical decision data. Our analyses revealed a facilitatory role of semantic transparency, with second constituent transparency playing a particularly robust facilitative effect on compound processing. We discuss these findings and their implications below.

### 5.1 Complementary strengths of computational estimates in capturing semantic transparency

Using computational models, including recent large language models (e.g., Brysbaert et al., 2025; Peng et al., 2025), for large‐scale estimates of psycholinguistic variables benefits from being less labor‐intensive. However, the extent to which computational estimates faithfully capture human semantic intuitions remains an open question.

In this study, we evaluated computational estimates against human ratings and found that different models show complementary strengths across the multidimensional construct of semantic transparency. Specifically, LLMs excelled at capturing compound‐level transparency, while traditional DSMs maintained advantages for constituent‐level transparency.

This difference likely reflects fundamental architectural and training distinctions between these approaches. LLMs, built on transformer architectures (e.g., Rogers et al., 2020; Vaswani et al., 2017), are trained on massive corpora (typically trillions of tokens; e.g., DeepSeek‐AI, 2025; Bai et al., 2023) using objectives such as next‐token prediction (e.g., GPT‐4) or multitoken prediction (e.g., DeepSeek‐V3). Through this extensive training, LLMs acquire rich statistical regularities and implicit linguistic knowledge that extend well beyond the local co‐occurrence patterns captured by traditional DSMs. This broader knowledge base may enable them to better model more sophisticated and implicit compositional processes underlying compound formation. A potential concern with LLM evaluation is data contamination (e.g., Trott, 2024; Xu, Guan, Greene, & Kechadi, 2024), where models may have encountered similar compositionality datasets during training. However, our database includes a substantial portion of previously unpublished compounds (2088 from Zhou et al. (2026)), which mitigates this concern and strengthens the validity of our findings.

In contrast, traditional DSMs trained on comparably smaller corpora (typically 10–100 million words) primarily capture semantic relatedness through distributional patterns (e.g., Zhang, Warstadt, Li, & Bowman, 2020). This makes them effective at representing how individual constituents as single concepts are semantically related to the concept of the compound as a whole. However, DSM performance varies substantially across implementations. Count‐based models like Latent Semantic Analysis (henceforth LSA) often underperform in predicting behavioral data (e.g., Auch et al., 2020; Kim et al., 2019), likely due to inferior representation quality compared to prediction‐based models trained on sufficient data (e.g., Lenci et al., 2023; Lenci & Sahlgren, 2023; Turney & Pantel, 2010). In our study, pretrained embeddings from TencentAI and fastText demonstrated robust performance (Table 3), while BERT‐based representations showed weaker results, possibly due to our limited sampling strategy (*N* = 10 sentences per concept). More systematic probing would be valuable in future work (e.g., Miletic & Schulte im Walde, 2023; Vulić et al., 2020).

### 5.2 Semantic transparency as a multidimensional construct

Given the different strengths of computational estimates across compound‐ and constituent‐level transparency, we investigated whether these measures, together with human ratings, necessarily capture the same underlying construct by examining their latent structure.

Our factor analysis of 27 transparency measures revealed a three‐dimensional latent structure distinguishing first constituent contribution (Factor 3), second constituent contribution (Factor 2), and overall compound predictability (Factor 1). This structure aligns with theoretical distinctions between constituent‐ and compound‐level conceptualizations of transparency (Gagné et al., 2019; Libben, 1998; Marelli & Luzzatti, 2012).

The emergence of separate factors for each constituent demonstrates that no single measure can fully capture their distinct contributions they provide to compound meaning. Although compound‐level transparency inherently involves interactions between both constituents, it emerged as a distinct dimension, as evidenced by the clustering of compound‐level measures (ratingCOMP, comp‐llm, comp‐com) on Factor 1. These findings provide robust support for the multidimensional nature of semantic transparency (Auch et al., 2020; Gagné et al., 2019).

The latent structure of semantic transparency in Chinese compounds partially aligns with Auch et al. (2020), who identified constituent position as the primary differentiator among English transparency measures. They also found that computational estimates formed a separate factor from human ratings. However, our analysis did not show strong evidence for such clear separation between computational estimates and human judgments, especially considering the substantial convergence observed between LLM‐generated and human ratings of compound‐level transparency. This divergence from prior findings likely reflects our broader range of computational methods and, more importantly, the use of more advanced language models (compared to earlier LSA‐based approaches used in previous studies). Despite these methodological differences, the finding that constituent position drives dimensional structure remains consistent across languages.

We also observed cross‐loadings between dimensions, which may reveal systematic interactions among constituent contribution and overall predictability. In our analysis, both constituents showed positive associations with overall compound predictability, with the first constituent (i.e., usually the modifier) demonstrating particularly robust loadings on Factor 1. Similar cross‐loading patterns between first constituent contribution and overall predictability were also observed in Auch et al. (2020). Such entanglement likely depends on their well‐described role in relation selection and semantic integration (Gagné & Shoben, 1997; Gagné & Spalding, 2013). For instance, in art.eps (“book bag”), the modifier art.eps (“book”) specifies the semantic relation to the head art.eps (“bag”) through an implicit *HEAD for MODIFIER* relation, facilitating interpretation of the compound's overall meaning.

### 5.3 Revisiting semantic transparency in compound processing: A multidimensional perspective

In this study, we also re‐examined the role of semantic transparency in Chinese compound processing. Specifically, we used factor scores as composite predictors, instead of relying on individual measures, to explain lexical decision performance. Our analyses revealed a facilitatory effect of semantic transparency, with the second constituent contribution (Factor 2) emerging as the most robust predictor of lexical decision performance.

The observed predictive prominence of the second constituent likely reflects the structural properties of Chinese compounds. As predominantly right‐headed (Chao, 1968; Song et al., 2022; Zhu, 1982), the second constituent typically serves as the semantic head, determining both semantic and grammatical properties of the compound. Transparent heads may thus provide more direct cues to compound meaning during lexical decision tasks, facilitating faster and more accurate processing (e.g., Libben et al., 2003; Marelli & Luzzatti, 2012). This pattern parallels findings from Auch et al. (2020), where factor scores related to the second constituent in English compounds more robustly predicted lexical decision times in both ELP and BLP datasets, while the first constituent effects were less robust across different analyses.

Notably, we did not observe a reliable effect for the first constituent, whereas previous Chinese compound processing studies reported significant effects for both constituents (e.g., Tse et al., 2017; Tsang, Zou, & Tse, 2022). One possible reason is that our relatively modest item size (*N* = 308) may have limited statistical power to detect a potentially smaller effect of the first constituent.^16^ Moreover, neurophysiological evidence from Tsang and Zou (2022) suggests that the second constituent exerts a more robust and sustained effect during the critical window for lexical access (100–400 ms), whereas the first constituent produces only a transient effect at an extremely early stage (0–100 ms) that diminishes before re‐emerging later (600–800 ms) as a late reanalysis phase. Since lexical decision times reflect overall processing duration rather than temporally specific neural responses, the second constituent's sustained effect may be more readily detectable in behavioral data. Future research with larger datasets could further elucidate the differential contributions of both constituents to compound processing.

In summary, these findings extend the multidimensionality hypothesis, previously established for English and German (e.g., Auch et al., 2020; Günther et al., 2020), to a typologically distinct language, Chinese. Additionally, our dataset, with its rich set of human ratings and computational estimates, provides a valuable resource for future research on compound representation and processing.

## Data Availability

Data and code are available at https://osf.io/wznce/.

## Notes

1 The wordlist from Zhou et al. (2026) is now also accessible through Qiu, Chersoni, Zhou, and Hsu (2026).

2
https://corpus.njau.edu.cn

3 Constituent segmentation was determined through a preliminary pilot study in which three Chinese Linguistics participants independently segmented each compound and then reached consensus on appropriate divisions.

4 A brief explanation with an example was provided: “In linguistics, the head of a compound is the constituent that determines the grammatical category and meaning of the entire compound. For example, in the compound  (‘go‐kart’, kart + car/vehicle), the head is  (‘car, vehicle'), indicating that the compound refers to a type of vehicle. The other constituent,  (‘kart'), specifies the type of vehicle.”

5 Following Wang et al. (2019), we normalized our ratings to the range [0,1] to eliminate rating scale differences.

6
https://modelscope.cn/models/lili666/text2vec‐word2vec‐tencent‐chinese/summary

7
https://huggingface.co/shibing624/text2vec‐word2vec‐tencent‐chinese/tree/main

8
https://fasttext.cc/docs/en/crawl‐vectors.html

9
https://huggingface.co/bert‐base‐chinese

10
https://huggingface.co/datasets/wikimedia/wikipedia

11 For targets with fewer than 10 sentences in the corpus, all available instances were included in the analysis.

12 The analysis included 2661 NNCs for which BERT representations of both constituents and compounds were successfully extracted. The remaining 14 compounds were excluded due to missing instances in the zhwiki‐20241101 dataset.

13 We extracted representations from the last layer of BERT for subsequent analysis.

14 For LLMs, we used averaged ratings across four iterations for subsequent analysis.

15
*factor_analyzer* package in *Python* was used: https://pypi.org/project/factor‐analyzer/.

16 We thank one reviewer for raising this point.
